# Synthesis of Diblock copolymer poly-3-hydroxybutyrate -*block*-poly-3-hydroxyhexanoate [PHB-*b*-PHHx] by a *β*-oxidation weakened *Pseudomonas putida* KT2442

**DOI:** 10.1186/1475-2859-11-44

**Published:** 2012-04-05

**Authors:** Lakshmi Tripathi, Lin-Ping Wu, Jinchun Chen, Guo-Qiang Chen

**Affiliations:** 1 MOE Key Lab of Bioinformatics, Department of Biological Science and Biotechnology, School of Life Science, Tsinghua-Peking Center for Life Sciences, Tsinghua University, Beijing, 100084, China; 2 Center for Nano and Micro Mechanics, Tsinghua University, Beijing, 100084, China; 3 Department of Pharmaceutics and Analytical Chemistry, Faculty of Pharmaceutical Sciences, University of Copenhagen, Copenhagen, Denmark

**Keywords:** Polyhydroxyalkanoates, PHB, Block copolymer, *Pseudomonas putida*, 3-hydroxybutyrate, 3-hydroxyhexanoate, Synthetic biology

## Abstract

**Background:**

Block polyhydroxyalkanoates (PHA) were reported to be resistant against polymer aging that negatively affects polymer properties. Recently, more and more attempts have been directed to make PHA block copolymers. Diblock copolymers PHB-*b*-PHHx consisting of poly-3-hydroxybutyrate (PHB) block covalently bonded with poly-3-hydroxyhexanoate (PHHx) block were for the first time produced successfully by a recombinant *Pseudomonas putida* KT2442 with its *β*-oxidation cycle deleted to its maximum.

**Results:**

The chloroform extracted polymers were characterized by nuclear magnetic resonance (NMR), thermo- and mechanical analysis. NMR confirmed the existence of diblock copolymers consisting of 58 mol% PHB as the short chain length block with 42 mol% PHHx as the medium chain length block. The block copolymers had two glass transition temperatures (*T*_*g*_) at 2.7°C and −16.4°C, one melting temperature (*T*_*m*_) at 172.1°C and one cool crystallization temperature (*T*_*c*_) at 69.1°C as revealed by differential scanning calorimetry (DSC), respectively. This is the first microbial short-chain-length (scl) and medium-chain-length (mcl) PHA block copolymer reported.

**Conclusions:**

It is possible to produce PHA block copolymers of various kinds using the recombinant *Pseudomonas putida* KT2442 with its *β*-oxidation cycle deleted to its maximum. In comparison to a random copolymer poly-3-hydroxybutyrate-*co*-3-hydroxyhexanoate (P(HB-*co*-HHx)) and a blend sample of PHB and PHHx, the PHB-*b*-PHHx showed improved structural related mechanical properties.

## Background

Polyhydroxyalkanoates (PHA) [1] are natural thermoplastics synthesized by many microorganisms as an intracellular storage material under unbalanced conditions of growth. As a substitute, to synthetic petrochemical polymers as well as to fulfill the growing demand of environmentally friendly plastics, production of PHA is under intensive studies [2]. PHA belong to a family of fully biodegradable polymers with no toxicity which can also be used for medical applications [3,4]. PHA can be completely bio-degraded into oligomers and monomers and then to CO_2_ and water, all are environmentally benign [5-7]. Some PHA have been developed for various applications including bioplastics for packaging, implant materials, biofuels and fine chemicals [8-10].

On the basis of monomer structures, PHA are divided into short chain length (scl) polymers consisting of monomers of C3-C5 carbon atoms, medium chain length (mcl) polymers consisting of monomers of C6-C16 carbons atoms, as well as PHA copolymers containing monomers of short chain length and medium chain length (scl-mcl) PHA [11-14]. The structures of PHA are decided by the specificity of a PHA synthase [15,16]. Poly-3-hydroxybutyrate (PHB), a scl PHA, has high crystalline nature with a low elongation to break and excessive brittleness [14]. Random copolymers of scl-mcl PHA such as poly(3-hydroxybutyrate-*co*-3-hydroxyhexanoate) (P(HB-*co*-HHx)), are considered to have better mechanical properties over scl copolymer poly(3-hydroxybutyrate-*co*-3-hydroxyvalerate) (PHBV) [14,17,18]. Copolymers of two or more monomers of scl or mcl are quite commonly produced, albeit they suffer from aging which affect their material properties [19,20].

Biosynthesis of homopolymers, random copolymers and block copolymers could be achieved by changing bacterial synthesis pathways and mode of cofeeding of substrates, during the bacterial cultivation process [21]. With the reduction of bacterial *β*-oxidation cycle, various types of PHA homopolymers were generated : genome reduced strains of *P. putida* KT2442, produced a series of homopolymers from C4-C7 and almost a homopolymer of C8 during the addition of structural related carbon substrates [22]. Homopolymers of C10 and C12 were produced by genome reduced *P. putida* derived strains [23,24]. Based on these results, PHA production can be designed to become a further platform for production of block copolymers using the genome reduced microbial strains which could produce desired PHA of the same chain length of fatty acids supplemented to the medium.

PHA with improved material properties have always been an important area of research [25]. To overcome the aging disadvantage of PHA, block copolymerization have been found to be a feasible way [21]. The properties of a polymer can be modified by introduction of different monomer units. Though, chemical synthesis of block copolymers is more common. Microbial production of block copolymers can be considered useful over chemically synthesized PHA as its biosynthesis can easily result in polymers with high molecular weights [26].

A recombinant and genome reduced bacterial strain can be constructed to produce different types of block copolymers either A-B (diblock), A-B-A (triblock) and (A-B)n repeating multiblocks [25]. Various types of PHA block copolymers such as PHB-*b*-PHBV [21], PHB-*b*-PHVHHp [27] and P3HB-*b*-P4HB [28] were reported. Yet block copolymerization of PHB and PHHx has not been yet achieved. Production of this diblock would allow to covalently bond scl and mcl PHA blocks together in order to achieve improved polymer properties.

*Pseudomonas putida* KT2442 consists of *pha* operon (*phaC1-phaZ-phaC2*) produces medium chain length PHA (C6-C14) [22]. *P. putida* KTOYO6 is a fatty acid *β*-oxidation impaired mutant in which 3-ketoacyl-CoA thiolase (*fadA*) and 3-hydroxyacyl-CoA dehydrogenase (*fadB*) genes were deleted to a maximum level in order to improve fatty acid utilization for PHA synthesis [29]. Since this strain is a derivative of *P. putida* it could only produce mcl PHA. *Aeromonas caviae* which harbors a PHA synthase operon *phaPCJ*_*Ac*_ could polymerize both scl and mcl monomers (C3-C7) [30,31]. To produce a scl-mcl PHA recombinant *P. putida* KTOYO6ΔC (*phaPCJ*_*Ac*_) was constructed by deletion of its PHA synthase genes and replaced by that from *Aeromonas caviae* (*phaPCJ*_*Ac*_). This recombinant strain was used to produce a block copolymer of PHB-*b*-PHVHHp [27]. The recombinant strain could further be utilized for the PHA block copolymer production due to its low substrate specific PHA synthesis enzymes *phaC*_*Ac*_*.*

In this study for the first time, with an attempt to prepare a PHA polymer better in properties than its random or blend polymer, we biosynthesized a diblock copolymer of poly(3-hydroxybutyrate)-*block*-poly(3-hydroxyhexanoate) (PHB-*b*-PHHx) using *P. putida* KTOYO6ΔC (*phaPCJ*_*Ac*_) strain. The diblock copolymer displayed better thermal and mechanical properties as expected over its random copolymer and blend sample of PHB and PHHx of similar composition. The detailed NMR comparison study proves the successful biosynthesis of the PHB-*b*-HHx.

## Results and discussion

*P. putida* KTOYO6ΔC (*phaPCJ*_*A.c*_) produced a polymer of 3-hydroxybutyrate (HB) or 3-hydroxyhexanoate (HHx) with minor amount of HB under addition of sodium butyrate and sodium hexanoate, respectively. Thus production of diblock poly-3-hydroxybutyrate-*block*-poly-3-hydroxyhexanoate (PHB-*b*-PHHx) under conditions of sequential addition of sodium butyrate and sodium hexanoate was achieved (Table 1).

**Table 1:** **Cell growth and PHA production by recombinant *P. putida* strains in the presence of fatty acids**

**Strain**	**Substrate**	**CDW(g/l)**	**PHA (wt %)**	**PHB (mol %)**	**PHHx (mol %)**
KTOYO6ΔC (*phaPCJ*_*A.c*_)	SB	3.71 ± 0.42	10.0 ± 0.31	100	0
	SH	2.44 ± 0.24	14.64 ± 0.67	13.82 ± 2.30	86.18 ± 4.65
	SB:SH (2:1)	4.75 ± 0.20	32.53 ± 0.74	74.35 ± 4.22	25.65 ± 3.27
	SB:SH (1:2)	5.82 ± 0.10	57.80 ± 1.12	57.70 ± 4.29	42.33 ± 5.26
KTQQ20	SH	1.67 ± 0.02	22.03 ± 0.42	0	100

SB: Sodium Butyrate, SH: Sodium hexanoate

SB:SH (2:1): 3 gL^-1^ SB was added at 0 h and 12 h during the cultivation process to form the PHB block After 24 h 3 g L^-1^ SH was added to form the PHHx block

SB:SH (1:2) : 3 gL^-1^ SB was added at 0 h and 3 g L^-1^ SH was fed at 12 h and 24 h of the cell growth.

Each shake flask process continued for 48 h. 20 g/l of glucose was fed at 0 h as a nutrient in each case of block formation. PHA samples were analyzed by GC [27]

All cells were grown in LB media for 48 h at 30 °C in the rotary shaker (HNY-2112B, Tianjin Honour Instrument Co. Ltd. China).

Wild type *Pseudomonas putida* KT2442 usually produces random mcl PHA [22]. During each *β*-oxidation cycle fatty acids lose two carbon atoms in the form of acetyl-coenzyme A (acetyl-CoA). Fatty acid degradation enzymes in *P. putida* including 3-ketoacyl-CoA thiolase and 3-hydroxyacyl-CoA dehydrogenase encoded by *fadA* and *fadB* respectively, are two important enzymes in the *β*-oxidation pathway [22,29]. Mutant *P. putida* KTOYO6 was constructed by deletion of 50% sequence of *fadA* and *fadB* in wild type *P. putida* KT2442. The reduction of *β*-oxidation genome prevented the shortening of fatty acid chain length thus, leading to the formation of PHA with the same carbon chain length as the length of the fatty acid supplement added in the media. The mutant strain accumulated more PHA as compared to the wild type [29]. To allow the production of diblock copolymers of a scl PHA block and a mcl PHA block, the *pha*C operon of *P. putida* KT2442 which favors mcl PHA synthesis, was replaced by an *Aeromonas caviae* (*phaPCJ*_*A.c*_) operon that is able to polymerize both scl and mcl monomers (C4-C7). The resulting mutant termed as *P. putida* KTOYO6ΔC (*phaPCJ*_*A.c*_) [27] was utilized in the present study.

### Microbial synthesis of PHA homopolymers and block copolymers

As observed in Table 1, recombinant *P. putida* KTOYO6ΔC (*phaPCJ*_*A.c*_) utilized sodium butyrate and sodium hexanoate for the formation of homopolymers PHB and PHHx containing small amount of HB, respectively. Pure homopolymer PHHx was produced by *Pseudomonas putida* KTQQ20, in which some essential *β*-oxidation genes were deleted, allowing the strain to produce mcl PHA of same carbon chain length as per the supplied fatty acids [23].

To produce PHB-*b*-PHHx, *P. putida* KTOYO6ΔC (*phaPCJ*_*A.c*_) was cultivated in LB media with glucose as a nutrient for cell growth. The contents and time of addition of the two carbon substrates in the form of sodium butyrate and sodium hexanoate were adjusted to achieve desired PHA (Table 1). When the two fatty acid substrates were added at an alternating time in the ratio of 2:1, 74 mol % PHB and 26 mol % PHHx accumulation took place. 3 gL^-1^ of sodium butyrate were fed at 0 h and 12 h, following the consumption of sodium butyrate as detected by the HPLC, after about 24 h, 3 gL^-1^ of sodium hexanoate was added during the 48 h of shake flask cultivation process . While, when the two fatty acids were supplemented in the ratio of 1:2 at different feeding times, PHA block composition consisted of 58 mol% PHB and 42 mol % PHHx. In this process of cultivation, 3 gL^-1^ of sodium butyrate was added at 0 h. After the consumption of sodium butyrate in the culture media, sodium hexanoate (3 gL^-1^) were added at 12 h and 24 h during total 48 h of shake flask cultivation (Table 1). Since the two cultivation methods described above produced same types of monomers only differing in PHA mol %, characterizations of only the putative PHB_58%_-*b*-PHHx_42%_ were performed. The PHB_58%_-*b*-PHHx _42%_ has been mentioned as PHB-*b*-PHHx unless stated otherwise. Thermal and mechanical characterizations as well as structure clarifications were carried out to confirm the putative block copolymerization.

### Physical characterization of the block copolymer

**Figure 1:**
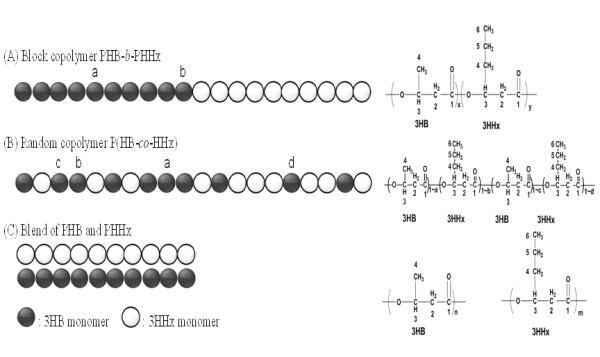
**Graphical representation of chemical structures of block copolymer PHB- *b* -PHHx (A), random copolymer P(HB- *co* -HHx) (B) and blend of PHB and PHHx (C).**

The putative block copolymer PHB-*b*-PHHx obtained after fractionation was analyzed and compared with a blend PHA containing homopolymers PHB and PHHx of the same compositions as that of the block copolymer and with a random copolymer of P(HB-*co*-HHx). Size exclusion chromatography (SEC) showed that the block copolymer had a number average molecular weight (*M*_n_) of 8.1 × 10^4^ and a polydispersity (PDI) of 2.5**.** NMR analysis was employed to elucidate the chemical microstructure [21,32,33] of the putative block copolymer PHB-*b*-PHHx, random copolymer P(HB-*co*-HHx) and blend of PHB and PHHx (Figure 1). Block copolymer PHB-*b*-PHHx is a special class of polymer that abruptly change from purely 3-hydroxybutyrate (3HB) monomer to purely 3-hydroxyhexanoate (3HHx) monomer (Figure 1A). This arrangement is very different from random copolymers, which maintain a constant average composition along the chain (Figure 1B) and blend of polymers does not have a chemical conjugation between 3HB monomer and 3HHx monomer (Figure 1C). In the blend of PHB and PHHx, monomers of 3HB and 3HHx exhibit only one chemical environment. In the putative block copolymer of PHB-*b*-PHHx, most of the 3HB and 3HHx monomers had one chemical environment, such as the 3HB unit *a* (Figure 1A-a), interacted among themselves which can expressed as B*B (B* B represents the interaction of monomer 3HB and 3HB, 3HB monomer abbreviated as B, and 3HHx abbreviated as X), except at the block changing point, the 3HB unit *b* (Figure 1A-b) had different chemical environment B*X. In the random copolymer of P(HB-*co*-HHx), the monomers of 3HB and 3HHx had four chemical micro-environments. For example in case of 3HB unit, 3HB monomer can be positioned between the 3HB unit or with 3HHx, which can be arranged as 3HB triad co-monomer sequence as following, B*B*B (Figure 1B-a), B*B*X (Figure 1B-b), X*B*B (Figure 1B-c), X*B*X (Figure 1B-d). At the same time, 3HHx monomer was similar as 3HB in its chemical micro-environments, and had four triad co-monomer sequences.

**Figure 2:**
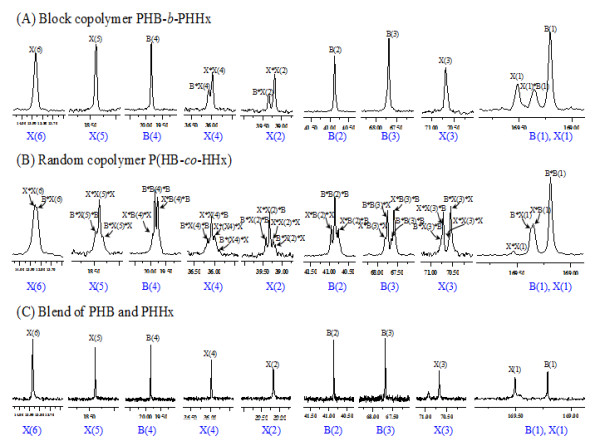
**Splittings of individual resonances of**^**13**^**C NMR spectra of block copolymer PHB- *b* -PHHx (A), random copolymer P(HB- *co* -HHx); (B) blend of PHB and PHHx(C); B and X refer to 3HB and 3HHx monomers, respectively**. The meaning of the numbering scheme can be referred to Figure 1. Chemical shifts are in ppm and tetramethylsilane (TMS) was employed as an internal chemical shift standard.

The NMR is a powerful tool for analyzing the detailed structures of copolymers. It can diagnose, not only the tacticity of PHA but also clearly differentiate, the PHA copolymer diad or triad sequence contribution based on the carbon resonances intensities topology in difference monomer sequences as also stated in previous studies [33-37]. In the putative block copolymer PHB-*b*-PHHx, most of the corresponding carbons in ^13^C NMR spectrum showed clear single peak resonances in each composition independently, there was no cross interrelation with each other except at the carboxyl group of 3HB(1), 3HHx(1), and methylene of 3HHx(2), 3HHx(4) which is near to the carboxyl group of 3HHx(1) (Figure 2A). In case of the random copolymer P(HB-*co*-HHx), all the carbon resonances of P(HB-*co*-HHx) were split into diad, triad, or quadruple, which reflects the sensitivity of the carbon nuclei to different chemical micro-environments of co-monomer sequences 3HB and 3HHx (Figure 2B). The assignments of the specific triad-monomer sequence for random copolymer P(HB-*co*-HHx) was cross-referenced to previous studies of random copolymer P(3HB-*co*-4HB) [38,39]. The relative peak intensities obtained from ^13^C NMR were interpreted in terms of co-monomer sequence distributions. The relative peak intensities obtained for each specific carbon resonance in 3HB and 3HHx units were used to determine the 3HB-centered or 3HHx-centered triad and diad sequence distributions. The carbonyl region B(l) and X(l), for example, comprised of four peaks, two singlets flanking a poorly resolved doublet; from left to right, these four peaks can be readily assigned to X*X(1), [X(1)*B, B(1)*X] and B*B(1) diad sequences. Of the remaining resonances, most showed lower chemical shift sensitivity to the co-monomer sequence. Interestingly, the main- and side-chain methylene carbon resonances of 3HHx, X(2) and X(4), were split into four peaks which can be assigned to the X centered triad co-monomer sequences X*X*XX*X*BB*X*XB*X*B. Peak assignments for X*X*X and B*X*B triads are unequivocal. However, those for B*X*X and X*X*B sequences were less certain. This co-monomer sequence was similar to random copolymer P(HB-*co*-HV) or PHBV.^37^ Compared with the ^13^C NMR spectrum of random copolymer P(HB-*co*-HHx), the responses of each carbon in the blend of PHB and PHHx were single and sharp since there was no interaction between 3HB monomer and 3HHx monomer (Figure 2C).

**Figure 3:**
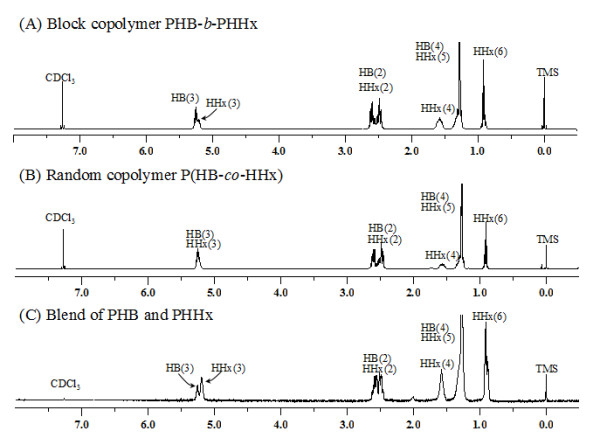
**The**^**1**^**H NMR spectra of block copolymer PHB- *b* -PHHx (A), random copolymer P(HB- *co* -HHx) (B) and blend of PHB and PHHx (C)**. The numbering scheme was same as that from Figure 1. Chemical shifts are in ppm and tetramethylsilane (TMS) was employed as an internal chemical shift standard.

Furthermore, in the spectrum of ^1^H NMR, clear separation of the hydrogen of 3HB(3) and 3HHx(3) in block polymer PHB-*b*-PHHx was observed while in the spectra of random copolymer P(HB-*co*-HHx) such separation was not found (Figure 3), since the 3HB and 3HHx monomers were randomly distributed in the micro-structure of random copolymer P(HB-*co*-HHx) chains. Spectra of the putative block copolymer PHB-*b*-HHx revealed a clear difference with random copolymer P(HB-*co*-HHx) and blend of PHB and PHHx. It confirmed the polymer to be the block copolymer PHB-*b*-PHHx consisting of PHB block covalently linked with PHHx block. Since biosynthesized PHA copolymers (either random or block copolymers) had a broad chemical compositional distribution as well as molecular weight distribution [40], fractionation processes were employed to separate the PHB-*b*-PHHx into various fractions to elucidate possible blends of PHB and PHHx mixed in the block copolymers of PHB-*b*-PHHx. As PHB typically exhibits early precipitation behaviors, it could be physically separated from its block or random copolymer, yet PHHx is an amorphous polymer that has the best solubility in organic solvents, it could not be easily precipitated by non-solvent addition method unless overdose of non-solvents were used. While, fractions containing block copolymers would exhibit properties of both polymers, it could be precipitated after PHB precipitation yet before that of the PHHx.

**Figure 4:**
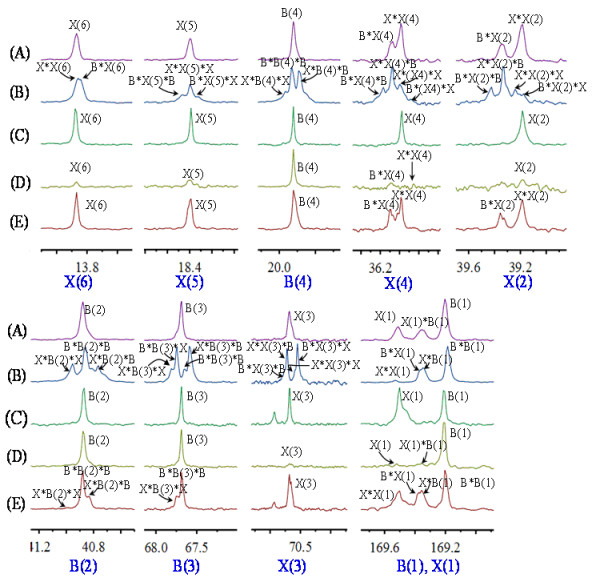
**Comparison of the splitting fingerprints of individual resonances of**^**13**^**C NMR spectra of the block copolymer PHB- *b* -PHHx (A), random copolymer P(HB- *co* -HHx) (B), blend of PHB and PHHx (C), the first fraction (F1) of PHB- *b* -PHHx (D), and second fraction (F2) of PHB- *b* -PHHx (E). B and X refer to 3HB and 3HHx, respectively; numbering scheme was the same as that in Figure**1. Chemical shifts are in ppm and tetramethylsilane (TMS) was employed as an internal chemical shift standard.

The fractions were examined via ^13^C NMR to determine the monomer sequences (Figure 4). The first fraction (F1) contained a polymer of 10.7 mol % PHHx and 89.3 mol % PHB as evidenced by ^13^C NMR which was sensitive enough to detect the PHHx signal, the carboxyl group of 3HB(1) and 3HHx(1) interacting with each other (Figure 4D), spectra of the F1 showed that the monomer sequence of 3HB and 3HHx existed as a block copolymer. The second fraction (F2) was a polymer consisting of 43.1 mol % 3HHx and 56.9 mol % 3HB, as revealed by the similarity of ^13^C NMR spectra of the block copolymer PHB-*b*-PHHx (Figure 2A). However, some unclear interactions of 3HB and 3HHx were observed (Figure 4E). The F2 carbon resonances of carbonyl region B(l) and X(l) were split into quadruple, indicating that F2 may have little amount of 3HB in the PHHx block. This detailed microstructure characterization of PHA fractions showed that the main sequence structures of the copolymers were block PHB-*b*-PHHx, while minor amount of PHB-*b*-P(HB-*co*-HHx) was also present in the whole polymer as also shown by the cell growth experiment (Table 1).

To further confirm the microstructure of the block copolymer, the diad-dependent carbonyl resonances in the ^13^ C NMR spectrum of the block copolymer PHB-*b*-PHHx and random copolymer P(HB-*co*-HHx) were split into multiple-peaks (Figure 2) with relative intensities and can be described by a Bernoullian model in Equation 1 [28,35]. If the polymer is a statistically random copolymer as described by the Bernoullian statistics, *F*_HHx*HHx_*, F*_HHx*HB_*, F*_HB*HHx_ and *F*_HB*HB_ (*F*_X*Y_ represents the molar fraction of *XY* sequence) can be expressed with the molar fraction of HHx *F*_*HHx*_ as follows:

(1) $$\begin{array}{c} F_{HHx*HHx}\;=\;{F_{\mathrm{HHx}}}^{2}\\ F_{HHx*HB}\;=\;F_{HB*HHx}\;=\;F_{\mathrm{HHx}}\left(1-F_{\mathrm{HHx}}\right)\\ F_{HB*HB}\;=\;{\left(1\;-F_{\mathrm{HHx}}\right)}^{2} \end{array}$$

In order to determine whether a polymer is a random copolymer or not, a parameter D is defined as follows:

(2) $$\text{D}\;=\;\left(F_{HHx*HHx}F_{HB*HB}\right)\;/\;\left(F_{HHx*HB}F_{HB*HHx}\right)$$

Analysis of the diad peaks revealed the nearest monomer neighbor distribution in the fraction, and the D statistic of Equation 2 was determined where *F*_HHx*HHx_ represents the fraction of 3HHx neighboring a 3HHx monomer, and so forth. Generally speaking, from Equation 2, random copolymers would have a D value near 1, the D value for a block copolymer should be much larger than 1 while that of an alternating copolymer should be smaller than 1 [32,41,42].

The fractions were examined with ^13^C NMR to determine the monomer sequence distributions. Analysis of fractions confirmed the block polymer PHB-*b*-PHHx microstructures according to ^13^C NMR spectrum (*F*_HHx*HHx_ = 0.2291, *F*_HB*HB_ = 0.1061, *F*_HB*HH x_ = 0.1061, *F*_HB*HHx_ = 0.5587) with a calculated D value of 11.37, which was much larger than 1. This provided further evidence that the sample was a block copolymer of PHB-*b*-PHHx. For the random copolymer of P(HB-*co*-HHx), *F*_HHx*HHx_ = 0.0303, *F*_HB*HB_ = 0.1273, *F*_HB*HHx_ = 0.2364 and *F*_HB*HHx_ = 0.6061 as calculated by the ^13^C NMR spectrum, these data led to a D value of 0.61, which was smaller than 1, confirming that this sample was a random copolymer of P(HB-*co*-HHx). In blend of PHB and PHHx, the monomers of 3HB and 3HHx had only one chemical environment, without cross-correlation with each other, their *F*_HB*HHx_ and *F*_HB*HHx_ were 0, its D value could not be calculated.

### Physical property characterizations of the block copolymer compared with its blend sample and random copolymer

**Figure 5:**
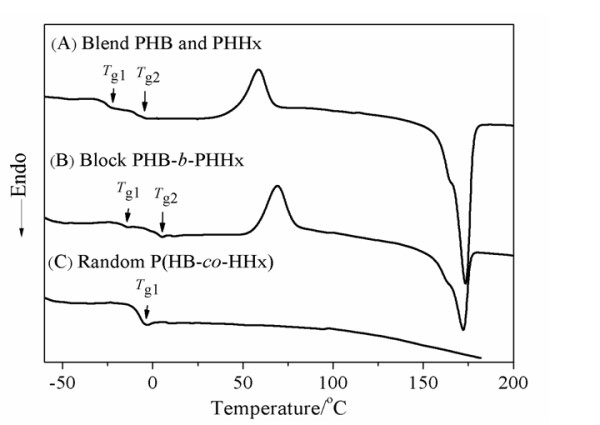
**DSC thermographs of the second heating process for the blend PHB and PHHx (A), block PHB- *b* -PHHx copolymer (B) and random P(HB- *co* -HHx) copolymer (C).**

Differential scanning calorimetry showed different thermal behaviours in different types of PHA as a result of variation in their chain structures [43] related to the block copolymer, blend sample of PHB and PHHx and random copolymer P(HB-*co*-HHx) (Figure 5). The block copolymer consisting of 42 mol % 3HHx displayed two glass transition temperatures (*T*_g_) at 2.7°C and −16.4°C (Table 2) which belong to PHB block segment and PHHx block segment, individually. Compared to homopolymer PHB and PHHx, *T*_g_ of the block copolymer PHB-*b*-PHHx were altered due to the interaction of independent polymer chains at the block changing point, the PHB block segment shifted to low *T*_g_, and the PHHx block segment shifted to high *T*_g_. Since the homopolymer PHHx did not possess any melting temperature, 172.1°C in the block copolymer indicates melting temperature of PHB chain segment. An obvious change was observed in the cooling crystallization temperature (*T*_c_) of the block copolymer compared with the *T*_c_ of blend PHB and PHHx, the *T*_c_ of block copolymer was 69.1°C while the *T*_c_ of blend PHB and PHHx was 58.6°C. In case of the PHB/PHHx blend, two *T*_g_ were found indicating that some part of PHB and PHHx were immiscible due to the lack of chemical conjugation between those two polymer segments. In a blend P(HB-*co*-HHx)/PHB consisting of 60 wt % P(HB-*co*-HHx), only one *T*_g_ valued at −5.15°C was observed which indicated a complete miscibility of P(HB-*co*-HHx) and PHB [43]. The random copolymer showed only one *T*_g_ due to the random assembly of chain segments.

**Table 2:** Comparison of physical properties of the block copolymer with different kinds of polymers

**PHA Type**	** *T* _**g**_ (°C)**	** *T* _**m**_ (°C)**	** *T* _**c**_ (°C)**	**Young’s** **Modulus** **(MPa)**	**Elongation** **At Break** **(%)**	**Tensile Strength** **(MPa)**
PHB ^*27*^	3.1	171.8	41.2	1470 ± 78.0	3.0 ± 0.40	18.0 ± 0.70
PHHx	−28.2					
Blend PHB/PHHx (PHHx 42 mol %)	- 8.2, -27.3	173.8	58.6	80.21 ± 5.23	10.14 ± 1.12	4.32 ± 0.45
Block PHB-*b*-PHHx (PHHx 42 mol %)	2.7, -16.14	172.1	69.1	7.58 ± 2.70	207.31 ± 15.38	1.42 ± 0.24
Random P(3HB-*co*-3HHx) (HHx 21 mol %)	−18.1	55.4		23.58 ± 4.10	75.29 ± 9.25	1.84 ± 0.36
Styrene butadiene rubber (SBR) ^*44,45*^	−55			1.80 ± 0.05	450.0 ± 0.0	2.10 ± 0.05

*T*_g_, glass transition temperature, *T*_m_, melting temperature, *T*_c_, crystallization temperature.

The mechanical properties of PHA block, blend and random sample were summarized in Table 2. The block copolymer PHB-*b*-PHHx had more elastomeric nature in comparison to its random and blend sample. The mechanical properties of the block copolymer were similar to that of natural rubber/elastomer (data not shown) or styrene butadiene rubber (SBR) containing about 23% styrene with low Young’s modulus and high elongation to break [44,45].

Finally, the differences in both thermo- and mechanical properties of PHB, PHHx, their blend and their putative block copolymers further confirmed the formation of the diblock copolymer PHB-*b*-PHHx. In summary, using the β-oxidation weakened *Pseudomonas putida* KTOYO6ΔC, for the first time, we were able to make short-chain-length PHB and medium-chain-length PHHx diblock copolymers.

## Conclusions

For the first time, short-chain-length PHB and medium-chain-length PHHx were microbially linked to form a diblock copolymer PHB-*b*-PHHx by a *β*-oxidation weakened *Pseudomonas putida* KTOYO6ΔC strain. The diblock structure was confirmed by NMR. Homopolymer PHB, a highly crystalline material, and homopolymer PHHx, an amorphous sticky material, both are not useful. Yet, block copolymerization based on the above two homopolymers, the PHB-*b*-PHHx, demonstrated some new and useful properties in comparison to its homo-, random and blend polymers. This block copolymer displayed dual properties of two individual homopolymer constituents covalently linked together, it had two *T*_g_ and had more flexible mechanical properties. The blends of homopolymers of PHB and PHHx are not miscible and random copolymers suffer from aging as reported by others. Block copolymerization opens a new area for PHA material property manipulation to meet specific applications.

## Methods

### Bacterial cultivation

#### Block copolymer production

The seed culture was prepared from 1% frozen culture of *P. putida* KTOYO6ΔC (*phaPCJ*_*Ac*_) frozen in 30% v/v glycerol and was inoculated into the LB medium at 30°C and 200 rpm for 12 h. A 5% v/v seed culture of *P. putida* KTOYO6ΔC (*phaPCJ*_*Ac*_) was grown in a 500 ml shake flask containing 100 ml LB of 5 g L^-^1 yeast extract, 10 g L^-1^ tryptone, 10 g L^-1^ sodium chloride and 20 g L^-1^ glucose. 50 mg L^-1^ kanamycin was added to maintain the plasmid stability at the beginning of cultivations. To promote the PHA accumulation, carbon sources in the form of fatty acids such as sodium butyrate and sodium hexanoate were added in required amount at subsequent intervals. The shake flask studies were performed for 48 h.

#### Homopolymer production

Recombinant *P. putida* KTOYO6ΔC (*phaPCJ*_*Ac*_) was used to produce homopolymer PHB on addition of sodium butyrate [27]. *P. putida* KTQQ20 (Δ*fadB2x,* Δ*fadAx,* Δ*fadB,* Δ*fadA,* Δ*phaG*) [23] derived from *P. putida* mutant weakened in β-oxidation pathway was utilized to produce homopolymer PHHx when sodium hexanoate was added. Seed cultures of *P. putida* KTQQ20 were prepared, approximately 5% volume of seed culture was inoculated into LB media in a 500 ml flask along with 100 mg L^-1^ ampicillin for plasmid maintenance. Sodium hexanoate with a concentration of 3 gL^-1^ was added after 12 h and 24 h, the shake flask studies were carried out for totally 48 h.

### PHA quantification

Bacterial cells were centrifuged at 9000 rpm for 5 min. The cell pellets were washed with ethanol and distilled water followed by lyophilization for 24 h. Cell Dry weight (CDW) was determined gravimetrically. PHA content and composition of lyophilized cells and extracted PHA were determined by gas chromatography (GC) using GC-2014 Shimadzu Gas Chromatograph [27].

Polymers were extracted from 5–10 g of dried cells dissolved in chloroform (wt/v) at 90°C for 4 h. The obtained supernatant was precipitated with 10 fold volume of cold ethanol. The precipitated PHA was dried under vacuum overnight.

The concentration of fatty acids were detected by high performance liquid chromatography (HPLC) (Spectra SYSTEM, SCM 1000) equipped with an organic analysis column (Aminex HPX-87 H ion column, 300 × 7.8 mm), an automatic sampler (SYSTEM AS3000), and a refractive index detector (Spectra SYSTEM, RI-150). A 50 mM H_2_SO_4_ was used as the mobile phase at a flow rate of 0.5 mL/min at 35°C.

### Fractionation of PHA

In order to confirm the structure of the putative block copolymer, the sample was fractionated with a chloroform/n-heptane solvent as previously described [32]. 1 g of PHA sample was dissolved in chloroform, and n-heptane was added to this solution until the PHA was precipitated at room temperature. After approximately 24 h the PHA suspension was centrifuged at 8000 g for 10 min. The precipitate was dissolved in a minimum amount of chloroform to form a polymer film. The film was subsequently dried at room temperature for 48 h. This process was repeated until no precipitate could be obtained on further addition of heptane. All fractionated polymers were analyzed by ^1^ H and ^13^C NMR (JOEL JNM- ECA 300, Japan) for the confirmation of putative block copolymer.

### Thermal characterization of PHA

Thermal characterization was performed using a Shimadzu DSC-60 differential scanning calorimeter (DSC). A sample of 2 mg in an aluminum-sealed pan was cooled from room temperature to −60°C by an auto cool accessory, and the pan was heated from −60°C to 180°C at a rate of 10°C min^-1^, isothermally maintained at 180° C for 3 min, quenched to −60°C, and reheated from −60°C to 180°C at a rate of 10 °C min^-1^ under a nitrogen flow rate of 50 ml min^-1^. Data was collected during the second heating run. The glass transition temperature (T_g_) was taken as the mid point of the heat capacity change, and the melting point (T_m_) was considered as the summit of melting peak. Cold crystallization temperature (T_c_) was determined from the DSC exothermal peak value and areas in the second scan.

### Nuclear magnetic resonance

The proton (^1^H) NMR was performed on JOEL JNM- ECA 300 NMR spectrophotometer in deuterated chloroform as a solvent, tetramethylsilane (TMS) was used as an internal chemical shift standard. Carbon (^13^C) NMR spectra was measured on 600 MHz spectrophotometer. ^1^H NMR provided the compositional value of 3HB and 3HHx monomer units and ^13^C NMR was used to determine the microstructure.

### Molecular weights and distribution

Molecular weights for the samples of PHA were analyzed on a size exclusion chromatography (SEC) at 40°C by a Waters 1525 pump with a combination of three styragel columns series (Styragel HR, 5 μm). A differential refractive index detector (2414, Waters, USA) were employed. Tetrahydro furan (THF) was used as an elution liquid at a rate of 1 mL min^-1^. Polystyrene standards (1.22 × 10^3^, 2.85 × 10^3^, 1.35 × 10^4^, 2.96 × 10_,_^4^ 1.97 × 10^5^ and 5.58 × 10^5^ in number-average molecular weights) with a low polydispersity were used to prepare a calibration curve.

### Mechanical properties

Mechanical property study was conducted as following [24]: Approximately 0.1 mm thick and 4 cm diameter PHA films were cast using chloroform as a solvent in flat bottom Petri dishes. The films were left to crystallize for approximately a week at room temperature. Subsequently, the films were cut into dumb bell shapes. The tensile mechanical property was studied on a Gotech AI-7000-S universal testing machine (Dong Guan, China) Co. Ltd. at room temperature at a speed of 5 mm min^-1^. P(HB-*co*-HHx) (HHx 21 mol %) were provided by Lukang Pharma Co. Ltd.(Shandong, China) as a gift to the lab for this study.

## Competing interests

All authors declare that they have no competing interests.

## Authors' contributions

LT performed most of the experiments and LPW analyzed the NMR data. JCC and GQC designed and supervised the studies. All authors read and approved the final manuscript.
